# Akt targeting as a strategy to boost chemotherapy efficacy in non-small cell lung cancer through metabolism suppression

**DOI:** 10.1038/srep45136

**Published:** 2017-03-23

**Authors:** Marion Le Grand, Raphael Berges, Eddy Pasquier, Marie-Pierre Montero, Laurence Borge, Alice Carrier, Sophie Vasseur, Veronique Bourgarel, Duje Buric, Nicolas André, Diane Braguer, Manon Carré

**Affiliations:** 1 Aix-Marseille Université, Inserm UMR_S 911, Centre de Recherche en Oncologie biologique et Oncopharmacologie, Faculté de pharmacie, Marseille, France; 2 Children’s Cancer Institute Australia, Lowy Cancer Research Centre, University of New South Wales, Randwick, NSW, Australia; 3 Metronomics Global Health Initiative, Marseille, France; 4 Centre de Recherche en Cancérologie de Marseille (CRCM), Aix-Marseille Université, Inserm U1068, Institut Paoli-Calmettes, 13009 Marseille, France; 5 Assistance Publique – Hopitaux de Marseille (AP-HM), Marseille, France; 6 Service d’Hématologie & Oncologie Pédiatrique, AP-HM, Marseille, France

## Abstract

Metabolic reprogramming is a hallmark of cancer development, mediated by genetic and epigenetic alterations that may be pharmacologically targeted. Among oncogenes, the kinase Akt is commonly overexpressed in tumors and favors glycolysis, providing a rationale for using Akt inhibitors. Here, we addressed the question of whether and how inhibiting Akt activity could improve therapy of non-small cell lung cancer (NSCLC) that represents more than 80% of all lung cancer cases. First, we demonstrated that Akt inhibitors interacted synergistically with Microtubule-Targeting Agents (MTAs) and specifically in cancer cell lines, including those resistant to chemotherapy agents and anti-EGFR targeted therapies. *In vivo*, we further revealed that the chronic administration of low-doses of paclitaxel - *i.e*. metronomic scheduling - and the anti-Akt perifosine was the most efficient and the best tolerated treatment against NSCLC. Regarding drug mechanism of action, perifosine potentiated the pro-apoptotic effects of paclitaxel, independently of cell cycle arrest, and combining paclitaxel/perifosine resulted in a sustained suppression of glycolytic and mitochondrial metabolism. This study points out that targeting cancer cell bioenergetics may represent a novel therapeutic avenue in NSCLC, and provides a strong foundation for future clinical trials of metronomic MTAs combined with Akt inhibitors.

Lung cancer remains the leading cause of tumor-related deaths worldwide. The most common form, non-small cell lung cancer (NSCLC) accounts for greater than 80% of all cases^1^. At the time of diagnosis, more than half of NSCLC patients present with locally advanced and/or metastatic disease, which confers a poor prognosis. Clinical treatment options are based on tumor histology and the presence of specific oncogenic drivers. Epidermal Growth Factor Receptor (EGFR) mutations and Anaplastic Lymphoma Kinase (ALK) rearrangements currently constitute the best-characterized molecular alterations in NSCLC associated with targeted therapies^2^,^3^. However, these two alterations are present in less than 50% of patients and resistance to targeted agents is frequent^4^. In this context, extensive efforts are ongoing to identify new therapeutic strategies.

Although cancer represents a heterogeneous group of diseases with a wide variety of genetic and epigenetic alterations, one common feature is the reprogramming of energy metabolism^5^. It is acknowledged that cancer metabolism is unique in the sense that most cancer cells avidly consume glucose, but they only use a small fraction for mitochondrial oxidative phosphorylation and tend to favor glycolysis even in the presence of oxygen^6^. This metabolic signature of cancer cells is not a passive response to damaged mitochondria but results from genetically-driven metabolic reprogramming^5^,^7^. Among known oncogenes, v-akt murine thymoma viral oncogene homolog (Akt) has been identified as a master regulator of the glycolytic pathway^8^. Many studies have characterized the abnormal activation of Akt in NSCLC as a frequent event, which correlates with a poor prognosis^9^,^10^,^11^. Therefore, targeted inhibition of Akt is an attractive therapeutic strategy as evidenced by the extensive list of Akt pathway inhibitors in development^12^,^13^,^14^. One of them, a third-generation alkylphospholipid called perifosine, is found to block Akt translocation to the membrane, thereby preventing Akt phosphorylation and activation^15^,^16^. Perifosine has shown promising anti-tumor activity in preclinical studies and clinical trials, especially when used in association with chemotherapy agents in different solid tumors and hematological malignancies^17^,^18^,^19^,^20^. A series of investigations have also highlighted its promising effect against NSCLC^21^,^22^,^23^.

Metronomic chemotherapy was first introduced in oncology 15 years ago and is defined as the chronic administration of chemotherapeutic drugs at relatively low, minimally toxic doses and with no prolonged drug-free breaks^24^. By relying on lower doses, metronomic chemotherapy decreases the toxicity of conventional cytotoxic drugs, thus providing a window of opportunity for combinatorial treatments with targeted agents. In a previous study, we disclosed a new signaling bridge between mitochondria and microtubules through Akt inhibition in the mechanism of action of Microtubule-Targeting Agents (MTAs)^25^. Indeed, we showed that the generation of mitochondrial ROS induced by MTAs was responsible for regulation of the Akt/GSK3β pathway that consequently triggered inhibition of microtubule dynamics, pointing out the need to re-examine the current dogma of microtubule targeting by MTAs. This work provided a rationale for the combination of MTA-based chemotherapy with Akt inhibitors. Herein, we report a synergistic anticancer activity of the combination of metronomic paclitaxel with perifosine. Furthermore, we unveil the underlying molecular mechanism *via* dual inhibition of glycolysis and mitochondrial bioenergetics in NSCLC. Our study therefore allows us to gain insights into the value of targeting energy metabolism to develop new anticancer strategies.

## Results

### Perifosine increases MTA efficacy selectively in cancer cells, in 2D and 3D culture models

**Figure 1:**
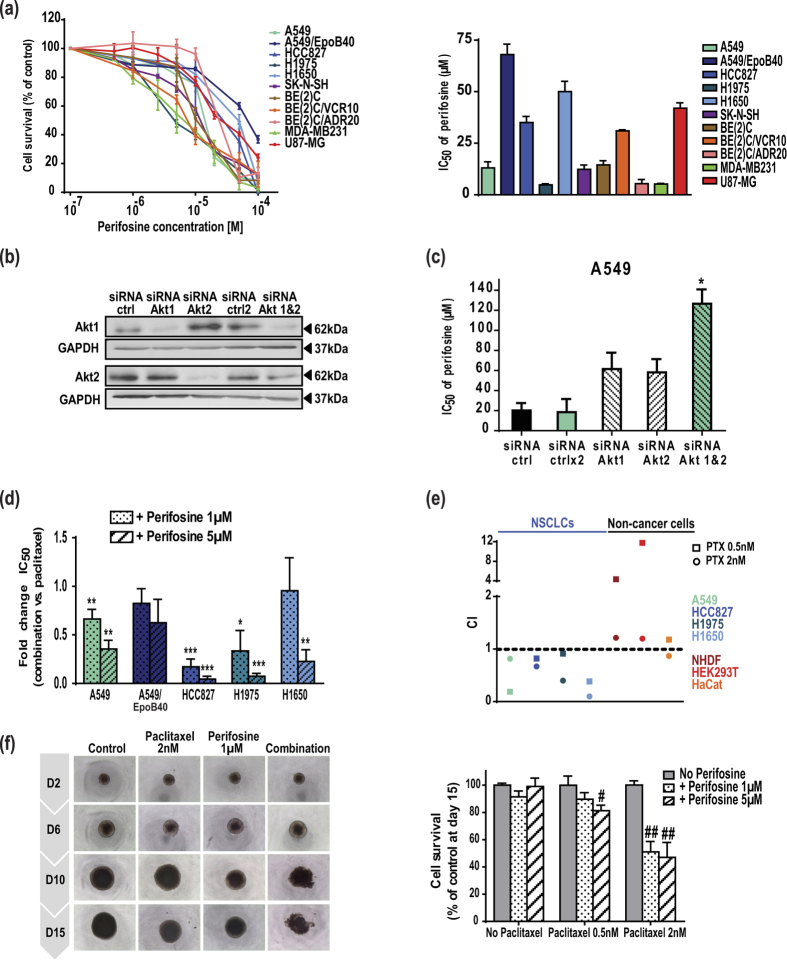
Perifosine increases MTA efficacy selectively in cancer cells, in 2D and 3D culture models through Akt inhibition. (**a**) Cell survival measured by MTT assay and IC_50_ of perifosine in a panel of eleven human cancer cells. (**b**) Levels of Akt1, Akt2 and GAPDH in A549 cells transfected with a control siRNA, Akt1 or Akt2 siRNAs. Samples derive from the same experiment and blots are processed in parallel. Uncropped blots are presented in Supplementary Fig. 4. (**c**) IC_50_ of perifosine in A549 cells transfected with a control siRNA, Akt1 or Akt2 siRNA. Significant differences compared to control siRNA. Mean ± S.E.M. of at least three independent experiments are shown. (**d**) Fold change in IC_50_ values of paclitaxel when combined to perifosine in NSCLC cells. Significant differences compared to IC_50_ of paclitaxel alone. (**e**) Dot plot representation of the CI of paclitaxel combined to perifosine 5 μM in NSCLC cells and non-cancer cells. (**f**) Representative pictures of A549 spheroids treated with paclitaxel and/or perifosine. Results were expressed as a percentage of growth in non-treated spheroids at day 15. Significant differences compared to paclitaxel treatment. Mean ± S.E.M. of at least three independent experiments are shown.

To evaluate the cytotoxic potential of perifosine, a panel of eleven human cancer cell lines was used (Supplementary Table 1). We showed that perifosine exerted dose-dependent cytotoxic effects against all cell types (IC_50_ values from 5 to 70 μM), including those resistant to conventional chemotherapies – MTAs and anthracycline – or to EGFR-targeted therapies (Fig. 1a). As a prerequisite for this study, we showed that exposition of NSCLC cells with perifosine decreased the active S473-phosphorylated form of Akt (Supplementary Fig. S1a). The use of Akt1 or Akt2 small interfering ribonucleic acid (siRNA) induced cell resistance to perifosine, definitely linking Akt targeting to perifosine efficacy (Fig. 1b,c). In agreement with the fact that perifosine is characterized as a pan-Akt inhibitor, the concomitant down-regulation of Akt1 and 2 led to a higher decrease in its efficacy. In addition, the dual down-regulation of Akt isoforms resulted in a 6-fold increase in cell response to paclitaxel, attesting to the pertinence of MTA/Akt inhibitor combination (Supplementary Fig. S1b). Thereafter, we carried out drug combination studies between perifosine and MTAs, *i.e*. paclitaxel or vincristine in line with clinical indications. Our data demonstrated that perifosine potentiated paclitaxel cytotoxicity in all the NSCLC cells tested (Supplementary Fig. S1c). This was reflected by a significant decrease in the IC_50_ values, including in the highly paclitaxel-resistant HCC827 cells pointing out that perifosine can, at least partially, bypass MTA resistance mechanisms (Fig. 1d). Moreover, the combination indexes (CI) indicated that the paclitaxel/perifosine treatment resulted in a synergistic interaction (CI < 1; Fig. 1e), even in the paclitaxel-resistant A549/EpoB40 cells (Supplementary Table 2). Similar trends were observed in the five NSCLC cells with perifosine at 1 or 10 μM, as well as with another Akt inhibitor LY294002 used as a reference inhibitor *in vitro* (data not shown). More importantly, similar synergisms were obtained in neuroblastoma, glioblastoma multiform and breast cancer cells, even when resistant to cytotoxic agents (Supplementary Fig. S2a,b). To limit secondary effects of treatment, we ensured that the synergism selectively occurred in cancer cells. Therefore, we showed that perifosine combined to paclitaxel or vincristine was in a majority antagonistic and at most additive in three human non-cancer cell types (Fig. 1e and Supplementary Fig. S2c and Table 1). Lastly, to recapitulate the cell-cell interactions within tumor masses and better anticipate response to drugs *in vivo*, we analyzed the efficacy of these combinations in 3D cell cultures. We demonstrated that combining paclitaxel with perifosine led to a significant decrease in A549 cell survival within the spheroids (Fig. 1f). As in 2D cell cultures, a significant reduction in cancer cell survival was also observed with the vincristine/perifosine combination in glioblastoma tumor spheroids (Supplementary Fig. S2d).

### Perifosine potentiates the anti-tumor efficacy of metronomic paclitaxel against lung adenocarcinoma *in vivo*

**Figure 2:**
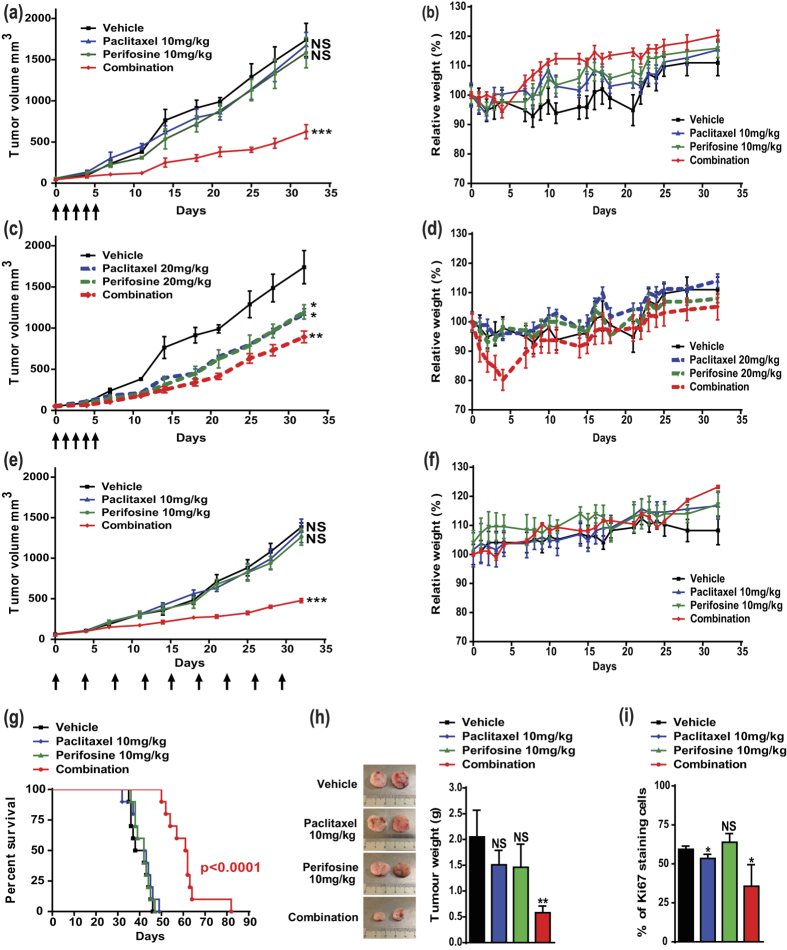
Metronomic paclitaxel/perifosine combination is highly effective in mice bearing human NSCLC xenografts. Perifosine was administered by oral gavage 5 days/week throughout the study (**a**–**f**), and paclitaxel was administered either once daily the first five days (**a**–**d**) or chronically twice a week throughout the study (**e**–**f**) as shown by arrows (n = 5). (**a**,**c**,**e**) Tumor volumes were measured. Significant differences compared to vehicle. (**b**,**d**,**f**) Weights of mice and (G) Kaplan-Meier survival curves (n = 10) from the same groups. (**h**) Representative pictures of tumors after dissection. Tumor weight was recorded. Significant differences compared to vehicle. (**i**) Immunohistochemical staining of the nuclear proliferation marker Ki67. Percentage of Ki67-positive cells with significant differences compared to vehicle.

To validate *in vivo* the promising results from the *in vitro* study, the paclitaxel/perifosine combination was investigated in mice bearing human NSCLC xenografts. A pilot study was conducted to determine optimal drug concentrations and schedule of treatment. Paclitaxel was first administered *i.v*. once daily for five consecutive days at 10 or 20 mg/kg. As expected, lower dose did not inhibit tumor progression (Fig. 2a) while higher dose reduced tumor growth by 33 ± 6% (Fig. 2c). Similar to paclitaxel, only higher dose of perifosine (20 mg/kg) had repressive effect on tumor growth (−31 ± 5%; Fig. 2c). Nevertheless, the Akt inhibitor significantly increased the anti-tumor efficacy of paclitaxel in all the combinations tested (Fig. 2a,c and Supplementary Fig. S3a,c). Interestingly, despite the lack of activity of the lower single-agent treatments, the combination of paclitaxel and perifosine at 10 mg/kg inhibited tumor growth more efficiently than the combination at higher doses (−64 ± 3% *versus* −49 ± 2%; *p* = 0.045; Fig. 2a and c). Furthermore, no major change in animal weight or other signs of potential toxicity were observed during the treatment with lower doses, while injection of paclitaxel at 20 mg/kg combined with perifosine led to an important weight loss in the first days of treatment (Fig. 2b,d and Supplementary Fig. S3b,d). These data clearly indicated that combining lower doses of paclitaxel and perifosine was both the most efficient and the best tolerated treatment schedule.

Lately, a growing body of evidence has emerged to highlight that metronomic treatment schedules can improve the efficacy of chemotherapy while minimizing toxic side effects^24^. To investigate the impact of Akt inhibition on metronomic chemotherapy, both paclitaxel and perifosine were administered chronically in mice bearing tumor at concentration of 10 mg/kg. Metronomic chemotherapy resulted in a reduction of tumor volume as pronounced as the standard treatment (−65 ± 1% *versus* −64 ± 3%; Fig. 2a and e) and was well tolerated (Fig. 2f), which allowed further analysis of the effects of metronomic paclitaxel and perifosine. As shown in Fig. 2g, median survival of mice treated with single agent was not different from the vehicle-treated mice, whereas the drug combination resulted in a significant increase in median survival (40 *versus* 61.5 days, *p* < 0.0001). Moreover, the administration of the two drugs at lower doses drastically reduced tumor weight (Fig. 2h). Compared to vehicle-treated tumors, the administration of each drug alone had no effect or only slightly reduced the percentage of Ki67 + cells. Consistently with the drop in tumor volume and weight, co-treatment of mice resulted in a decrease in the number of Ki67+ cells (−40 ± 13%; Fig. 2i and Supplementary Fig. S3e). Our *in vivo* study thus provided definitive evidence of the high efficacy of perifosine combined with paclitaxel, especially at low-doses in lung cancer.

### Low concentrations of perifosine enhance the pro-apoptotic effects of paclitaxel independently of cell cycle arrest

**Figure 3:**
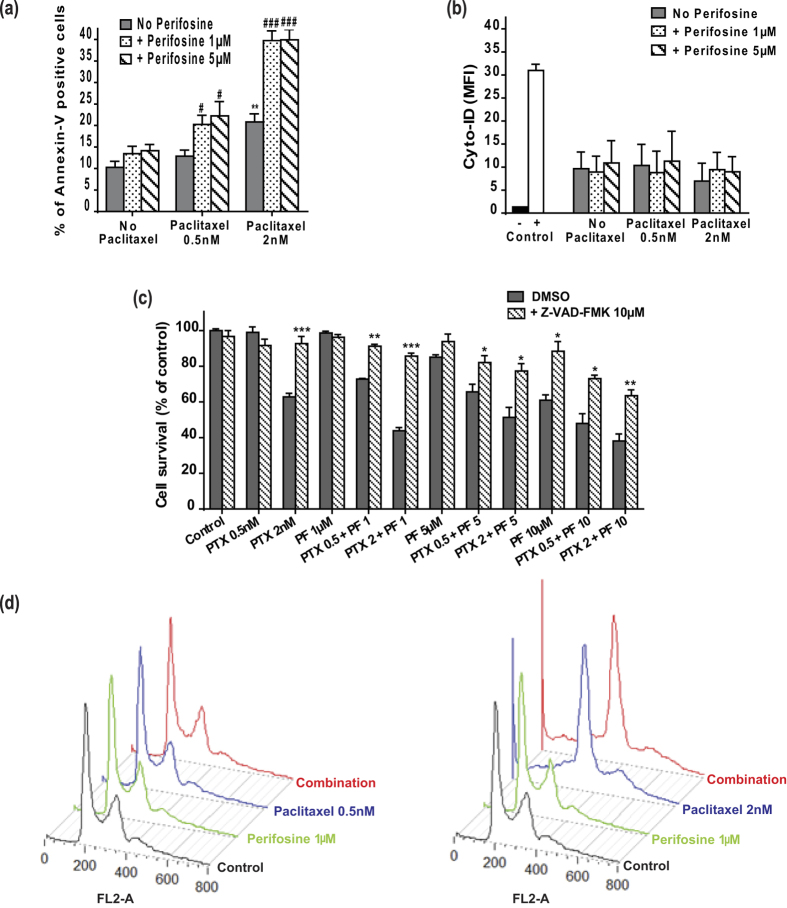
Low doses of perifosine enhance the pro-apoptotic activities of paclitaxel without modifying the paclitaxel-mediated cell cycle arrest. (**a**) Annexin-V positive A549 cells were quantified after paclitaxel and/or perifosine treatment. Significant differences compared to vehicle (*) or to paclitaxel alone (#). Mean ± S.E.M. of at least three independent experiments are shown (**b**) Quantification of Cyto-ID mean fluorescence intensity (MFI) in A549 cells treated with paclitaxel and/or perifosine. A549 cells treated with 5 μM rapamycin and 50 μM chloroquine were used as positive control and untreated A549 cells as negative control. (**c**) A549 cells were pre-treated with 10 μM of Z-VAD-FMK during 3 h before paclitaxel and/or perifosine treatment. Cell survival was revealed by the MTT assay. Significant differences compared to vehicle treatment. Mean ± S.E.M. of at least three independent experiments are shown (**d**) Representative cell cycle profiles of A549 cells exposed to paclitaxel and/or perifosine.

Given that MTAs are known to inhibit cancer progression by triggering programmed cell death^26^, we first analyzed whether perifosine could potentiate paclitaxel efficacy through apoptosis induction. Although perifosine low concentrations did not induce lung cancer cell death when used alone, it significantly potentiated paclitaxel-mediated apoptosis, as shown by the 2-fold increase in Annexin-V positive cells when exposed to paclitaxel 2 nM and perifosine 1 μM (Fig. 3a). In contrast, autophagy did not contribute to cell response to treatment (Fig. 3b). We further showed that the combined treatment induced cell death through a caspase-dependent signaling pathway, since pre-treating A549 cells with the pan-caspase inhibitor Z-VAD-FMK significantly increased cell survival, irrespective of the drug concentration used (Fig. 3c). Lastly, cell cycle analysis demonstrated that perifosine neither triggered nor enhanced the expected mitotic arrest when combined with paclitaxel (Fig. 3d). Collectively, our results showed that perifosine increased the pro-apoptotic activity of paclitaxel without potentiating its anti-mitotic properties in NSCLC cells.

### Perifosine exerts a dose-dependent anti-glycolytic effect in lung cancer cells

**Figure 4:**
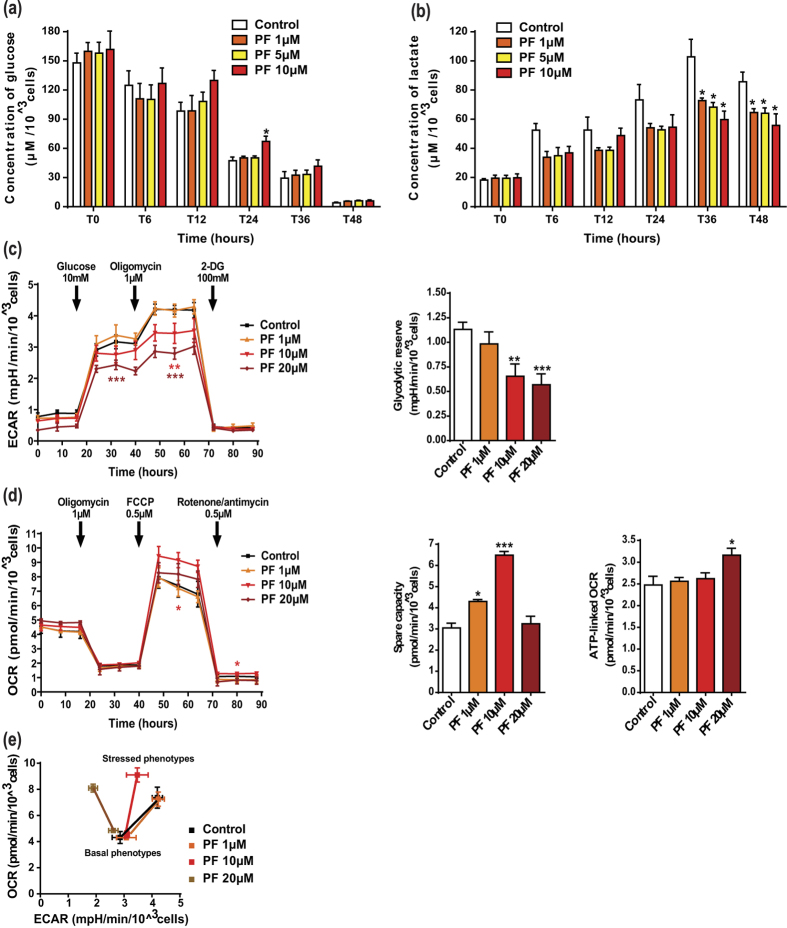
Perifosine decreases the glycolytic pathway in NSCLC cells. (**a**,**b**) Supernatants from A549 cells treated with perifosine (PF) were collected over time and analyzed for (**a**) glucose consumption and (**b**) lactate production by using the YSI 2900^®^ instrument. Analyte concentrations were normalized to cell number. Significant differences compared to control. (**c**–**e**) A549 cells treated with perifosine were analyzed for glycolysis and mitochondrial bioenergetics using the Seahorse XF^®^ technology. (**c**) Real-time measurement of ECAR in A549 cells. The glycolytic reserve was calculated as the difference between oligomycin-enhanced and glucose-mediated ECAR values. (**d**) Real-time measurement of OCR in A549 cells. Spare capacity was calculated as the difference between maximal and basal respiration, while ATP-linked OCR was the difference between basal and minimal values. ECAR and OCR were normalized to cell number. Significant differences compared to control. (**e**) Energy phenotypes of A549 cells after perifosine treatment. Mitochondrial respiration and glycolysis were first simultaneously evaluated under the starting medium conditions (baseline phenotypes) and then upon injection of FCCP and oligomycin respectively (stressed phenotypes). The metabolic potential (dotted lines) revealed cell preferred pathway to meet an energy demand. Mean ± S.E.M. of at least three independent experiments are shown.

Because accelerated glycolysis is a common feature of cancer cell survival^27^ and Akt has been identified as a glycolytic inducer^8^, we examined the changes that perifosine may exert on glycolysis. While only perifosine 10 μM significantly slowed down glucose consumption (at 24 hr), A549 lactate production was efficiently reduced (36 and 48 hr) by low and high concentrations of perifosine (Fig. 4a and b). To confirm the drug impact on lactate production, we additionally performed real-time measurements of extracellular acidification rate (ECAR). Perifosine-treated cells showed a decreased initial ECAR, confirming its anti-glycolytic properties (Fig. 4c left). When mitochondrial respiration was suppressed by the Adenosine Tri-Phosphate (ATP) synthase inhibitor oligomycin, glycolysis was stimulated to compensate the lack of energy production. It remained nevertheless at low levels in perifosine-treated cells, as reflected by the dose-dependent reduction in glycolytic reserve (Fig. 4c right). To characterize the effects of perifosine on mitochondrial respiration, we evaluated the oxygen consumption rate (OCR). Perifosine, regardless of the concentration, did not modify the basal OCR (Fig. 4d left). When the electron transport chain uncoupler carbonilcyanide p-trifluoromethoxyphenylhydrazone (FCCP) was injected, mitochondrial respiration was stimulated, mimicking an increase in energy demand and showing the maximal respiratory capacity. Unexpectedly, the spare capacity was enhanced by 1 and 10 μM of perifosine (Fig. 4d middle). It is noteworthy that the OCR-linked ATP generation was not simultaneously increased by treatment, excepted for the highest concentration (Fig. 4d right). Lastly, we represented the metabolic potential of A549 cells, which characterized their ability to respond to high energy demand *via* mitochondrial respiration and/or glycolysis (Fig. 4e). Our data showed that basal phenotypes after perifosine treatment were similar to those in non-treated cells. Interestingly, whereas control cells can accelerate the two bioenergetic pathways to reach a stressed phenotype, cells exposed to perifosine exhibited a dose-dependent inhibition of glycolysis, forcing them to exclusively use the mitochondrial respiration as indicated by the ECAR decrease associated with the OCR increase.

### Combining low concentrations of paclitaxel and perifosine results in a dual inhibition of mitochondrial and glycolytic metabolism

**Figure 5:**
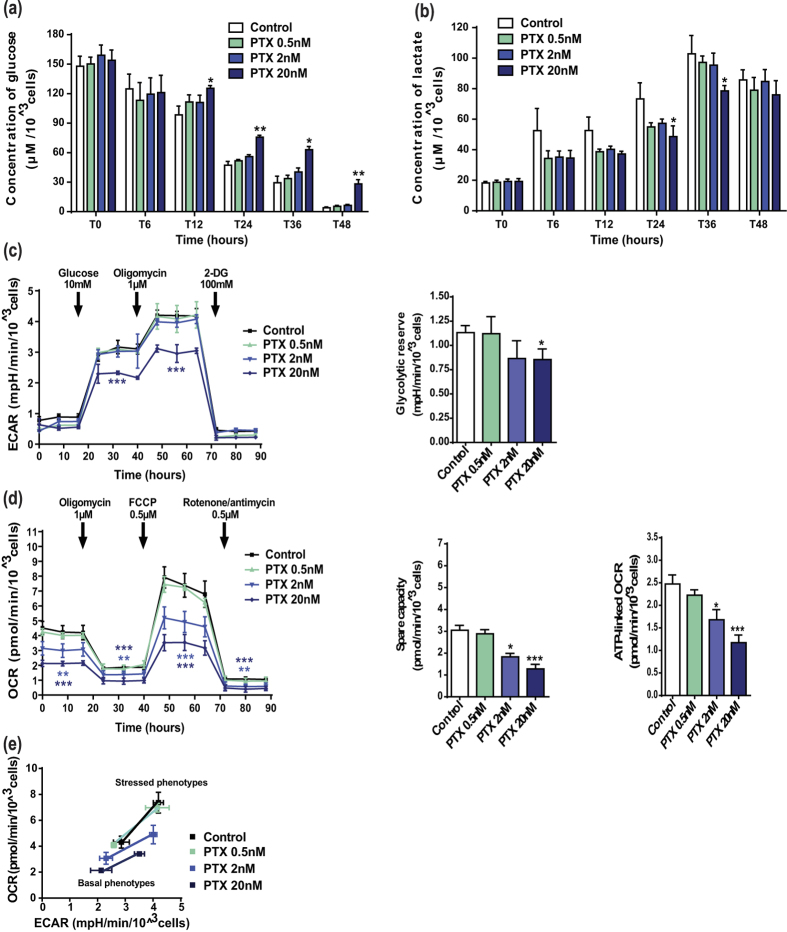
Paclitaxel has an anti-glycolytic component and inhibits the overall mitochondrial metabolic parameters in NSCLC cancer cells. (**a**,**b**) The kinetic measurement of glucose consumption and lactate production after paclitaxel (PTX) treatment was conducted as described in Fig. 4. (**c**,**d**) Experimental steps and data analysis of ECAR and OCR measurements in A549 cells exposed to paclitaxel followed the same procedure as those reported in Fig. 4. Significant differences compared to control. (**e**) Energy phenotype of A549 cells exposed to paclitaxel. Baseline and stressed phenotypes, as well as metabolic potential, were determined as defined in Fig. 4. Mean ± S.E.M. of at least three independent experiments are shown.

**Figure 6:**
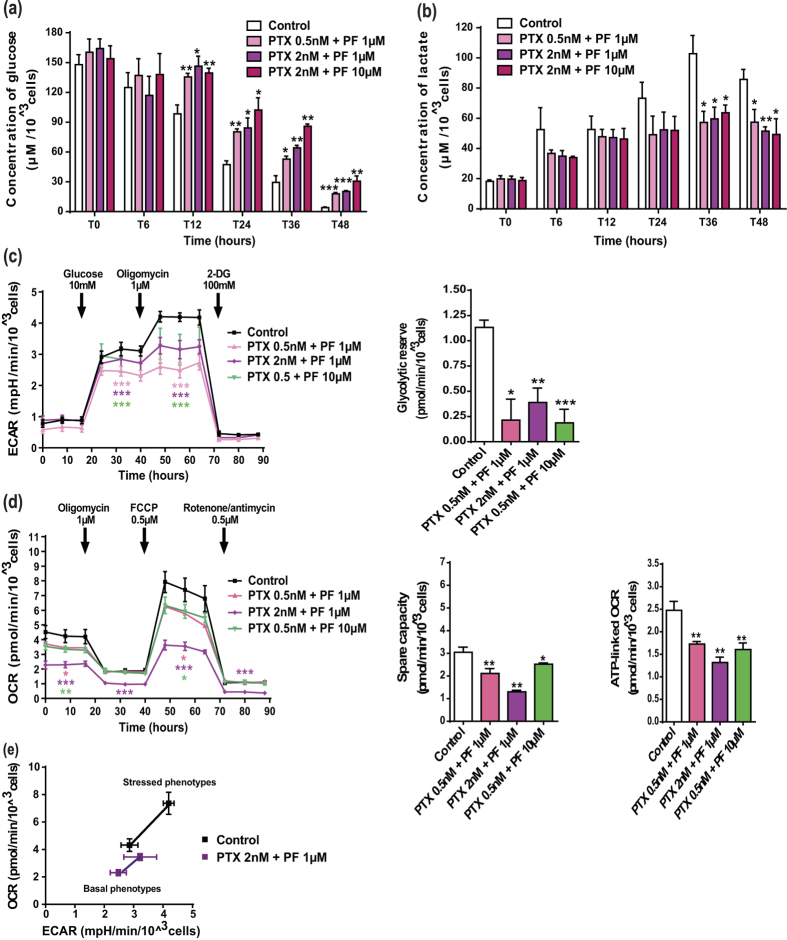
Low concentrations of paclitaxel with perifosine are able to impact the whole cellular metabolism in A549 cancer cells. (**a**,**b**) The kinetic measurement of glucose consumption and lactate production after paclitaxel (PTX) and perifosine (PF) treatment was conducted as described in Fig. 4. (**c**,**d**) Experimental steps and data analysis of ECAR and OCR in A549 cells exposed to paclitaxel/perifosine combination followed the same procedure as those reported in Fig. 4. Significant differences compared to control. (**e**) Energy phenotype of A549 cells exposed to the combination of 2 nM paclitaxel and 1 μM perifosine. Baseline and stressed phenotypes, as well as metabolic potential, were determined as defined in Fig. 4. Mean ± S.E.M. of at least three independent experiments are shown.

To elucidate the molecular mechanisms underlying the efficacy of this combination, we investigated how it affected the energetic metabolism. Firstly, we showed that A549 cells exhibited decreased glucose uptake and lactate production at high concentration of paclitaxel (20 nM; Fig. 5a and b). Consistently, only this high paclitaxel concentration led to a reduction in both the initial and maximal ECAR, as well as in the glycolytic reserve (Fig. 5c). However, it induced a dose-dependent decrease in the overall mitochondrial metabolic efficiency, as shown by a reduction of basal OCR, maximal OCR, spare respiratory capacity and OCR-linked ATP production (Fig. 5d). Moreover, representation of the cell metabolic potential after paclitaxel treatment mainly showed a dose-dependent inhibition of OCR of either basal or stressed phenotypes (Fig. 5e). More interestingly, combination of low concentrations of perifosine and paclitaxel, which had no metabolic activity when used alone, strongly affected the energetic pathways. For example, combining paclitaxel 2 nM and perifosine 1 μM significantly reduced glucose uptake and lactate release from A549 cells (Fig. 6a and b), as well as initial ECAR (−13 ± 2%), maximal ECAR (−24 ± 3%) and the glycolytic reserve (−65 ± 13% compared to control) (Fig. 6c). Similar conclusions were drawn with regards to the impact on glycolysis of the different concentrations tested (Fig. 6a–c). Moreover, the combinatorial treatment resulted also in a significant inhibition of OCR and related mitochondrial metabolic parameters, paclitaxel 2 nM and perifosine 1 μM being the most obvious example (Fig. 6d). Lastly, metabolic potential analysis of this combination confirmed that the drug co-administration led to a concomitant disruption of OCR and ECAR in cells of either basal or stressed phenotypes (Fig. 6e), which prevented the dynamic shift between glycolysis and mitochondrial respiration. In conclusion, our results clearly demonstrated that combining paclitaxel and perifosine provided a unique opportunity to efficiently impact cancer cell metabolism.

## Discussion

Despite recent advances in cancer treatments, lung cancer is still a lethal cancer in adult accounting for the most common cause of tumor-related death^1^. Alterations of the Akt signaling pathway in a variety of cancers, including NSCLC, and its involvement in chemo-resistance have instigated the development of Akt inhibitors^12^,^13^,^14^. When used as monotherapy, these molecules were associated with toxicity and minimal efficacy. They have nevertheless demonstrated promising anti-tumor activity in association with anticancer drugs^19^,^20^. Such combinatorial approach has yet to be assessed in lung cancer. Our previous work highlighted the key role of Akt in cancer cell response to MTAs, providing a strong rationale for MTA/anti-Akt combination^25^. In the present study, we used Akt siRNA and the pan-Akt inhibitor perifosine to demonstrate increased sensitivity to MTAs associated with Akt inhibition in NSCLC cells. Based on its efficacy in our 2D and 3D models of glioblastoma multiforme, neuroblastoma or breast cancer, the relevance of the MTA/anti-Akt combination may be widened to other refractory cancers. Lastly, the antagonistic effects observed in non-cancer cells reflected treatment selectivity, definitely supporting the pertinence of combining MTA-based chemotherapy with Akt inhibitors.

Acquired resistance to EGFR-targeted therapies regularly occurs in patients who initially respond to treatment^4^. Mechanisms leading to resistance include hyperactivation of the downstream Phosphoinositide 3-kinase (PI3K)/Akt pathway^28^. Herein, we showed that, whether used alone or in combination, perifosine was active in cells that did not respond to EGFR-targeted therapies, pointing out the ability of anti-Akt to bypass this resistance mechanism. Consistent with our results, a recent phase II trial of erlotinib (anti-EGFR) plus MK-2206 (anti-Akt), conducted in patients with advanced NSCLC that previously progressed on erlotinib, provided proof of feasibility for Akt pathway inhibition to overcome acquired resistance to anti-EGFR agents^29^. In recent years, studies have also highlighted the potential of perifosine to restore cancer cell sensitivity to conventional cytotoxic agents, such as etoposide in neuroblastoma cells^18^. Our work demonstrated that perifosine sensitized chemo-resistant NSCLC cells to paclitaxel and neuroblastoma cells to vincristine, supporting further evaluation of this therapeutic approach.

In our study, the synergism observed between perifosine and paclitaxel *in vitro* translated into increased anti-tumor effects *in vivo* and resulted in prolonged median survival in NSCLC xenograft-bearing mice. We found that the metronomic treatment schedule was very well tolerated and highly efficient in reducing tumor volume. Several reports recently suggested that metronomic chemotherapy could increase the efficacy of targeted therapies without drastically intensifying their side effects^24^. Metronomically-administered MTAs have been clinically evaluated in NSCLC in combination with targeted agents such as Vascular Endothelial Growth Factor (VEGF) or EGFR inhibitors^30^,^31^, but never with anti-Akt compounds. Therefore, our preclinical data provided strong foundation for future clinical trials that could evaluate the safety and efficacy of metronomic MTAs combined with Akt inhibitor in lung cancer.

To optimize anti-cancer treatment schedules, it is crucial to gain insights into drug mechanism of action. We highlighted that perifosine did not affect NSCLC cell cycle progression when used alone, nor potentiated the mitotic arrest induced by paclitaxel when used in combination. This may reflect cell-type dependent molecular consequences of Akt inhibition, since previous data reported cell cycle arrest induced by perifosine in medulloblastoma and hepatocellular carcinoma^32^,^33^. In NSCLC, the efficacy of the paclitaxel/perifosine combination mainly relied on the induction of a caspase-dependent apoptotic cell death. This may be related to perifosine-mediated suppression of Akt pro-survival properties, which were described to result from apoptotic regulator inhibition and decrease in transcription factor activity^34^. Lastly, while studies showed that perifosine can induce protective autophagy in leukemia and glioblastoma cells^35^,^36^, our data clearly demonstrated that it did not influence NSCLC cell response to perifosine alone or in combination with chemotherapy.

The field of energy metabolism in cancer biology is expanding fast, and metabolic regulation is emerging as a strategy of choice to control tumor progression^27^. The metabolic adaptation of cancer cells was initially linked to mitochondrial respiration dysfunction^6^. However, it is now clear that mitochondria generally retain full capacities and can be reactivated when high energy levels are required^37^. This suggests that cancer cell metabolic activity results from reversible changes that could be targeted pharmacologically to hinder tumor development^38^. In this study, we revealed that perifosine displayed potent glycolysis inhibitory properties. We further showed that Akt inhibitor forced NSCLC cells to exclusively use the mitochondria as their energy source, leading to an increase in mitochondrial ATP production at high perifosine concentrations. This metabolic reprogramming could explain why Akt inhibitor lacks single-agent activity in different human cancers^39^,^40^,^41^. In support of this, the metabolic shift toward mitochondrial bioenergetics has been recently identified as being responsible for glioblastoma cancer cell resistance to molecules that target PI3K, the major activator of Akt^42^. To overcome this metabolic adaptation, a concomitant inhibition of the glycolytic energetic pathway and the mitochondrial respiratory machinery has emerged as a promising strategy to efficiently trigger cancer cell death^43^,^44^. Here, we demonstrated that the efficacy of the paclitaxel/perifosine combination, especially at low concentrations, was linked to an inhibition of the two major bioenergetic pathways.

To conclude, the synergistic combination of metronomic paclitaxel and perifosine is a promising therapeutic strategy. Our work highlights the potential of this type of rational combination to improve response to chemotherapy and overcome drug resistance in NSCLCs, by targeting energy metabolism. In the future, more research should be dedicated to identifying predictive biomarkers of response to select patients who are likely to benefit from this combination therapy.

## Material and Methods

### Cell culture

The human NSCLC cells A549, HCC827, H1650 and H1975, as well as MDA-MB231 breast cancer cells, U87-MG glioblastoma cells, SK-N-SH neuroblastoma cells, and the human HEK293T epithelial embryonic kidney cells, primary cultures of HaCat keratinocytes and normal dermal NHDF fibroblasts were purchased from ATCC cell bank (Manassas, USA). A549/EpoB40 NSCLC cells were a gift from Dr. SB Horwitz (Albert Einstein College of Medicine, NY, USA). BE(2)C, BE(2)C/VCR10 and BE(2)C/ADR20 neuroblastoma cells were kindly provided by Pr. M. Kavallaris (Children’s Cancer Institute, Sydney, Australia). Routine culture media and cell resistance to conventional chemotherapy agents and targeted therapies are included in Supplemental Table 1.

### Drugs and reagents

Stock solution of paclitaxel (Novasep synthesis), LY294002 (Life Technologies, France) and Z-VAD-FMK (Invitrogen) were prepared in dimethyl sulfoxide (DMSO) while vincristine (Lilly, France) was prepared in sterile distilled water. Perifosine, purchased from Abcam (UK) for *in vitro* studies and from Selleckchem (USA) for *in vivo* studies, was prepared in phosphate-buffered saline (PBS). The highest concentration of DMSO cells were exposed to was 0.001%.

### Cytotoxicity test

After a 72 h treatment, cell survival was measured by the (3-(4,5-dimethylthiazol-2-yl)-2,5-diphenyl tetrazolium bromide) MTT assay (Sigma-Aldrich) as we previously performed^25^. The combination index (CI) values were calculated with the Calcusyn software^®^ according to the Chou and Talalay theorem, which sets out the additive effects (CI = 1), synergisms (CI < 1) and antagonisms (CI > 1) in drug combination.

### Spheroid outgrowth assay

Cells were seeded in 96-well round bottom plates with medium containing 20% methylcellulose (Sigma-Aldrich) for 48 h. Spheroids were then treated three times per week during 15 days. Images were daily captured with an Olympus microscope (10×/N.A. 0.45 objective lens). Spheroid growth inhibition at day 15 was determined using 10 μl of Alamar blue for 24 h, and absorbance was measured at 510–580 nm with a PolarStar plate reader (BMG Labtech).

### Transfection study

A549 cells were transfected with siRNA Akt1 (Flexitube siRNA Hs Akt1 11 SI02758406, Qiagen, Germany), siRNA Akt2 (Flexitube siRNA Hs Akt2 6 SI0299173, Qiagen) or negative siRNA control (Allstars neg Control siRNA 1027781, Qiagen) using Lipofectamine 2000 (Invitrogen) according to the protocol supplied by the manufacturer.

### Western blot analysis

Cells were lysed in radioimmunoprecipitation assay buffer (RIPA, Tris 50 mM pH 8.0, NaCl 250 mM, Triton-X100 0.1%) and western blot was performed as previously described^25^. The primary antibodies used were directed against α-tubulin (clone DM1A, Sigma Aldrich, 1/1000), pAkt-S473, Akt-total (Cell Signaling, USA, 1/1000) or glyceraldehyde 3-phosphate deshydrogenase (GAPDH, Sigma Aldrich, 1/10000). Peroxydase-conjugated secondary antibodies (Jackson Immunoresearch, USA) and chemiluminescence detection kit (Millipore) were used for visualization. Signal was quantified with Image J^®^ software.

### Flow cytometry

Flow cytometry analysis of cell cycle and apoptosis, in cells treated for 24 h or 48 h respectively, was performed as previously described^45^. For autophagy analysis, cells were treated for 24 h, harvested and incubated for 30 min at 37 °C with 1:500 Cyto-ID^®^ autophagy detection reagents (Enzo Life Sciences, Enz-51031-K200). Flow cytometry analysis was performed with BD FACSCalubur™ (BD Biosciences) and data were analyzed in BD CellQuest™ Pro software.

### Real-time metabolic analysis

Simultaneous multiparameter metabolic analysis of live cells was performed in the Seahorse XF24^®^ extracellular flux analyzer (Seahorse Bioscience, USA). A549 cells were seeded in XF24 V7 multi-well plates (15,000 cells per well) for 5 h at 37 °C in 5% CO_2_. Cells were then exposed to drugs for 24 h prior to the assay. One hour before recording the glycolytic activity, cell culture medium was replaced with minimal DMEM (0 mM glucose) without phenol red supplemented with 143 mM NaCl, 2 mM glutamine and 1 mM sodium pyruvate, pH 7.4. Extracellular acidification rate (ECAR) was measured under these basal conditions and after sequential injections of glucose (10 mM), of the ATP synthase inhibitor oligomycin (1 μM), and of the glycolysis inhibitor 2-deoxyglucose (100 mM). To record the mitochondrial activity, the same assay medium was used and supplemented with 1 mM sodium pyruvate and 10 mM glucose. Oxygen consumption rate (OCR) was analyzed before and after sequential injections of oligomycin (1 μM), of the electron transport chain uncoupler FCCP (1 μM) and of specifc inhibitors of the mitochondrial respiratory chain antimycin A/rotenone (0.5 μM). To normalize OCR and ECAR data to cell number, cells were fixed with glutaraldehyde 1%, stained with violet crystal 0.1% in methanol 20% (Sigma-Aldrich), which was finally solubilized in DMSO to measure dye absorption with a microplate spectrophotometer (600 nm).

### Glucose consumption and lactate production assay

YSI 2950^***®***^ (Life Sciences, USA) was used to measure the total flux of glucose and lactate. Cells were exposed to drugs 24 h after seeding. At each time point, medium was collected, centrifuged 5 min at 1,200 rpm and kept at −20 °C before measurement. Metabolite concentrations were normalized to cell number as described for real-time metabolic analysis.

### *In vivo* animal model

All the procedures and animal care complied with French Government official guidelines. *In vivo* experiments were approved (authorization number 0100903) by the Regional Committee for Ethics on Animal Experiments (Marseille number 14), and were conducted in the Scientific Centre for Functional Exploration (approval number F-13-055-5). Female BALB/c nude mice 6 to 8 weeks old were obtained from Charles Rivers Laboratories (France). 5 × 10^6^ A549 cells were inoculated subcutaneously into the right dorsal flanks of the nude mice. Experimental treatments were started when tumor reached about 50 mm^3^ in volume (day 0). Initial pilot studies (n = 5) comprised administration of paclitaxel either by consecutive daily intravenous (i.v.) injections for 5 days or twice a week until experiment completion. It was dissolved in ethanol/cremophor EL (1:1) solution and given at 10 or 20 mg/kg, i.e. one or two thirds of the maximum tolerable dose (MTD, 28 mg/kg). Injections (10 mL/kg) were administered through the tail vein after intraperitoneal anesthesia with xylazine (10 mg/kg) and ketamine (100 mg/kg). For each schedule protocol, perifosine, dissolved in sterile PBS, was given by oral gavage 5 days/week at 10 or 20 mg/kg, i.e. one or two thirds of the MTD (30 mg/kg). Weight of mice was daily determined. Twice a week, tumor volumes were determined by caliper measurements, according to the formula Length × Width × Depth × 0.5236. For the survival study, mice (n = 10) were allowed to live until their natural death or were sacrificed when their tumor volume exceeded 2,000 mm^3^. Survival medians were estimated by the Kaplan-Meier product limit method. The log-rank test was used to compare survival rates by univariate analysis.

### Immunostaining

Tumors were excised, weighed and sectioned. For each subcutaneous tumor, 4 μm thick paraffin sections were prepared and stained with Ki-67 (Ventana Medical Systems, France). Staining was performed using the Benchmark XT automate (Ventana Medical Systems) according to the manufacturer’s instructions.

### Statistical analysis

Each experiment was performed at least in triplicate. Data are presented as mean ± S.E.M. Statistical significance was tested using unpaired Student’s t test. For experiment using multiple variables, statistical significance was assessed *via* two-way ANOVA. A significant difference between two conditions was recorded for *p < 0.05; **p < 0.01; ***p < 0.001. Statistical analyses were performed with GraphPad 5.0 statistical software.

## Additional Information

**How to cite this article:** Le Grand, M. *et al*. Akt targeting as a strategy to boost chemotherapy efficacy in non-small cell lung cancer through metabolism suppression. *Sci. Rep.* **7**, 45136; doi: 10.1038/srep45136 (2017).

**Publisher's note:** Springer Nature remains neutral with regard to jurisdictional claims in published maps and institutional affiliations.

## Supplementary Material

Supplementary Data
