# Supplementary material for: Akt targeting as a strategy to boost chemotherapy efficacy in non-small cell lung cancer through metabolism suppression
Source: Sci Rep. 2017 Mar 23;7:45136. doi: 10.1038/srep45136 (PMC5362809; doi:10.1038/srep45136)
Supplement: Supplementary Data [file srep45136-s1.pdf]

Akt targeting as a strategy to boost chemotherapy efficacy in non-small cell lung cancer through metabolism suppression.

Marion Le Grand[1,2], Raphael Berges[1], Eddy Pasquier[1,3], Marie-Pierre Montero[1], Laurence Borge[4], Alice Carrier[4], Sophie Vasseur[4], Veronique Bourgarel[1,5], Duje Buric[1], Nicolas André[1,3,6], Diane Braguer[1,5] and Manon Carré[1,*].

[1] Aix-Marseille Université, Inserm UMR_S 911, Centre de Recherche en Oncologie biologique et Oncopharmacologie, Faculté de pharmacie, Marseille, France
[2] Children's Cancer Institute Australia, Lowy Cancer Research Centre, University of New South Wales, Randwick, NSW, Australia
[3] Metronomics Global Health Initiative, Marseille, France
[4] Centre de Recherche en Cancérologie de Marseille (CRCM), Aix-Marseille Université, Inserm U1068, Institut Paoli-Calmettes, 13009 Marseille, France
[5] Assistance Publique – Hopitaux de Marseille (AP-HM), Marseille, France
[6] Service d'Hématologie & Oncologie Pédiatrique, AP-HM, Marseille, France

* to whom correspondence should be addressed:
Manon Carré, PhD
Centre de Recherche en Oncologie biologique et Oncopharmacologie
Faculté de Pharmacie
27 boulevard Jean Moulin
13385 Marseille Cedex 05
France
Phone number: 33 (0)4-91-83-56-26
Email: manon.carre@univ-amu.fr

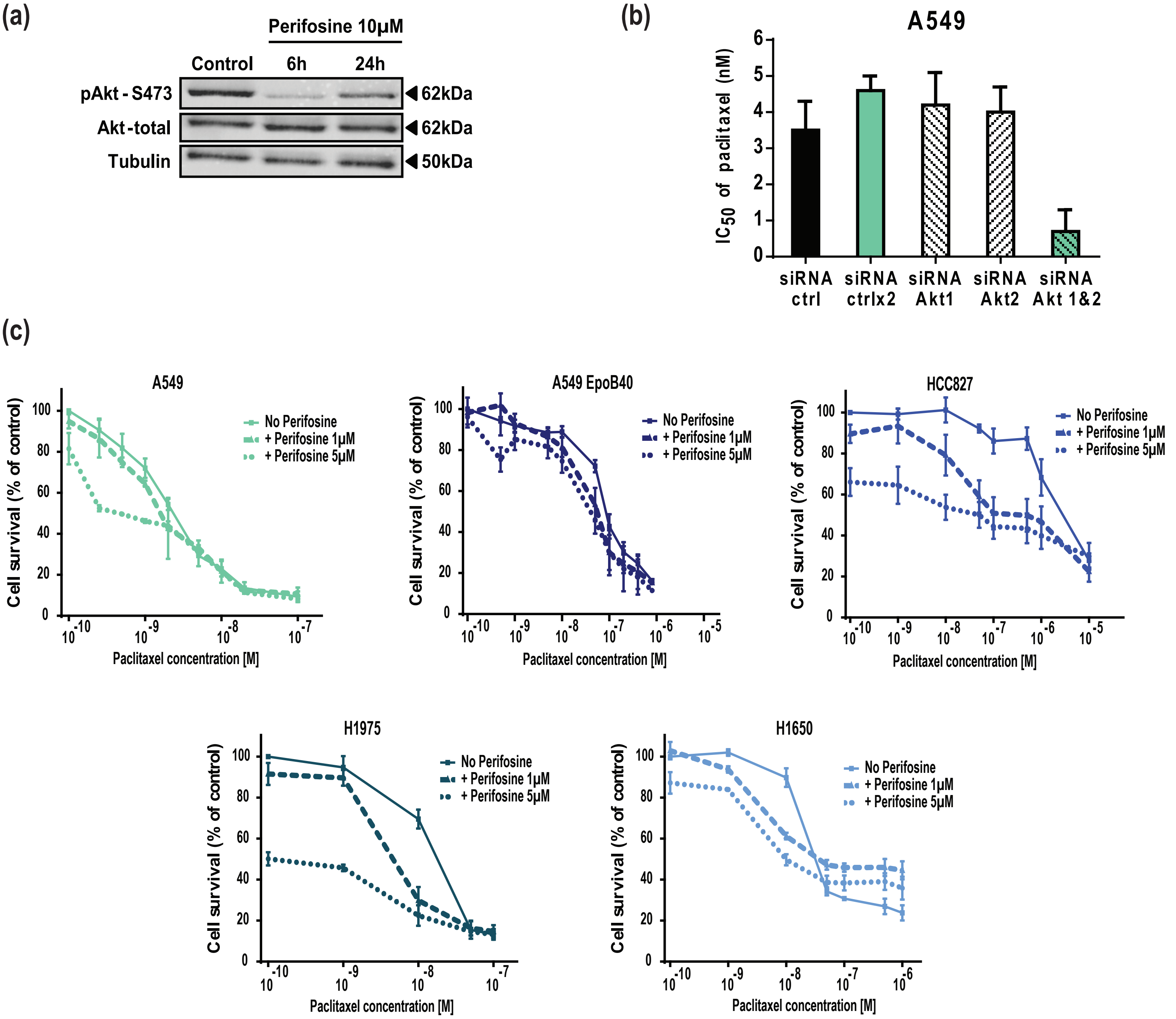
(a)
Perifosine 10μM
Control
6h
24h
pAkt - S473
Akt-total
Tubulin
62kDa
62kDa
50kDa
(b)
A549
IC50 of paclitaxel (nM)
siRNA ctrl
siRNA ctrlx2
siRNA Akt1
siRNA Akt2
siRNA Akt 1&2
(c)
A549
A549 EpoB40
HCC827
H1975
H1650
No Perifosine
+ Perifosine 1μM
+ Perifosine 5μM
Cell survival (% of control)
Paclitaxel concentration [M]

Supplementary Figure 1: (a) Levels of active pAkt-S473, Akt-total and tubulin in A549 cells after a 6 h and 24 h-treatment with 10 μM perifosine. (b) IC_{50} of paclitaxel in A549 cells transfected with a control siRNA, Akt1 or Akt2 siRNAs. (c) Cell survival after paclitaxel treatment alone or combined to perifosine 1 or 5 μM in NSCLC cells. Mean ± S.E.M of at least three independent experiments are shown.

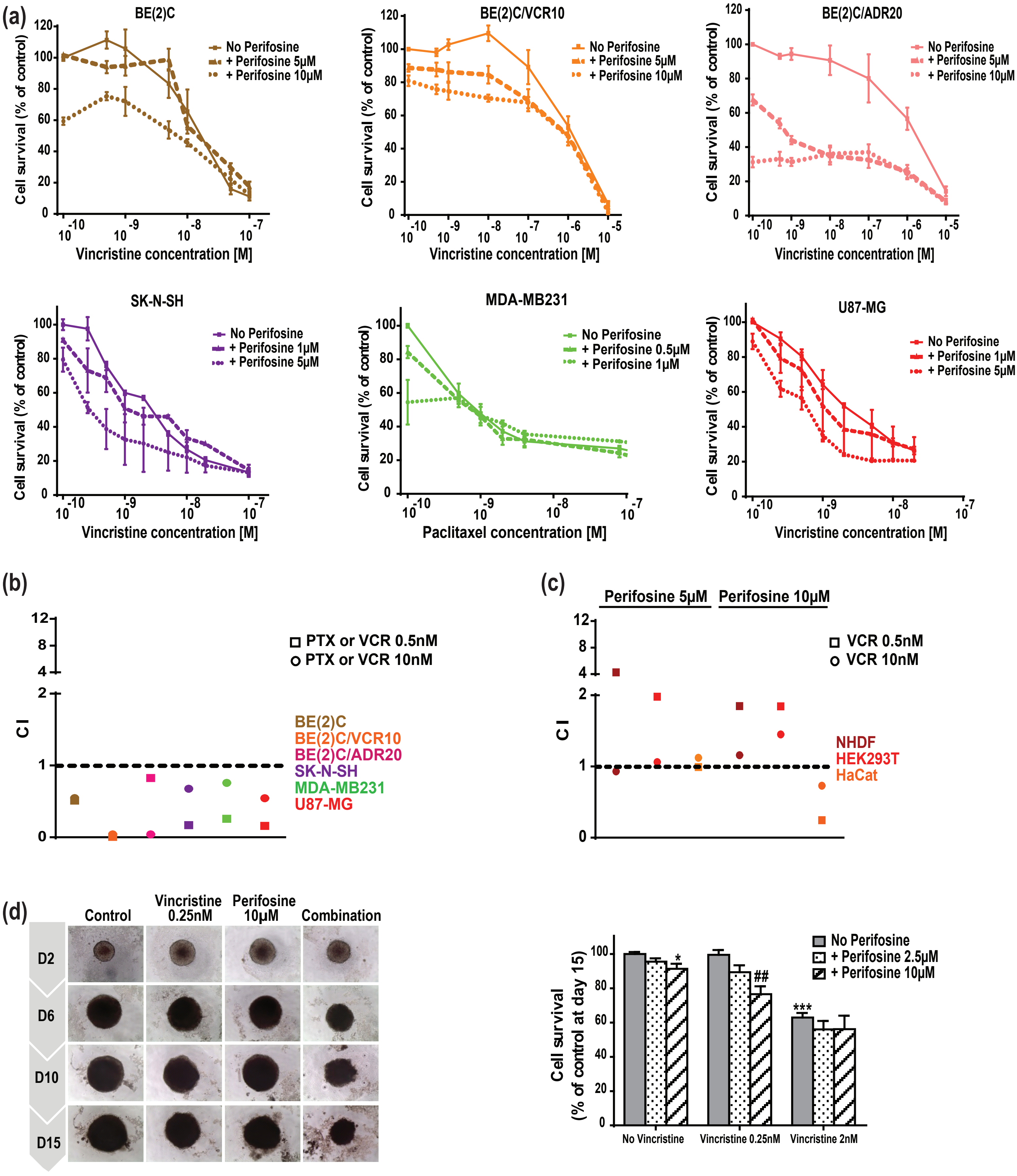
(a)
BE(2)C
No Perifosine
+ Perifosine 5µM
+ Perifosine 10µM
Cell survival (% of control)
Vincristine concentration [M]
BE(2)C/VCR10
No Perifosine
+ Perifosine 5µM
+ Perifosine 10µM
BE(2)C/ADR20
No Perifosine
+ Perifosine 5µM
+ Perifosine 10µM
SK-N-SH
No Perifosine
+ Perifosine 1µM
+ Perifosine 5µM
MDA-MB231
No Perifosine
+ Perifosine 0.5µM
+ Perifosine 1µM
Paclitaxel concentration [M]
U87-MG
No Perifosine
+ Perifosine 1µM
+ Perifosine 5µM
(b)
CI
PTX or VCR 0.5nM
PTX or VCR 10nM
BE(2)C
BE(2)C/VCR10
BE(2)C/ADR20
SK-N-SH
MDA-MB231
U87-MG
(c)
Perifosine 5µM
Perifosine 10µM
VCR 0.5nM
VCR 10nM
NHDF
HEK293T
HaCat
(d)
Control
Vincristine 0.25nM
Perifosine 10µM
Combination
D2
D6
D10
D15
Cell survival (% of control at day 15)
No Perifosine
+ Perifosine 2.5µM
+ Perifosine 10µM
No Vincristine
Vincristine 0.25nM
Vincristine 2nM

Supplementary Figure 2: (a) Cell survival after paclitaxel or vincristine treatment alone or combined to perifosine at indicated concentrations in different cancer cells. (b) Dot plot representation of the CI of paclitaxel or vincristine combined to perifosine 5 µM in different cancer cells. (c) Dot plot representation of the CI of vincristine combined to perifosine in three human non-cancer cell types. (d) Representative pictures of U87-MG spheroids treated with vincristine and/or perifosine. Results were expressed as a percentage of growth in non-treated spheroids at day 15. Significant differences compared to no treatment condition (*) or to vincristine treatment (#). Mean ± S.E.M of at least three independent experiments are shown.

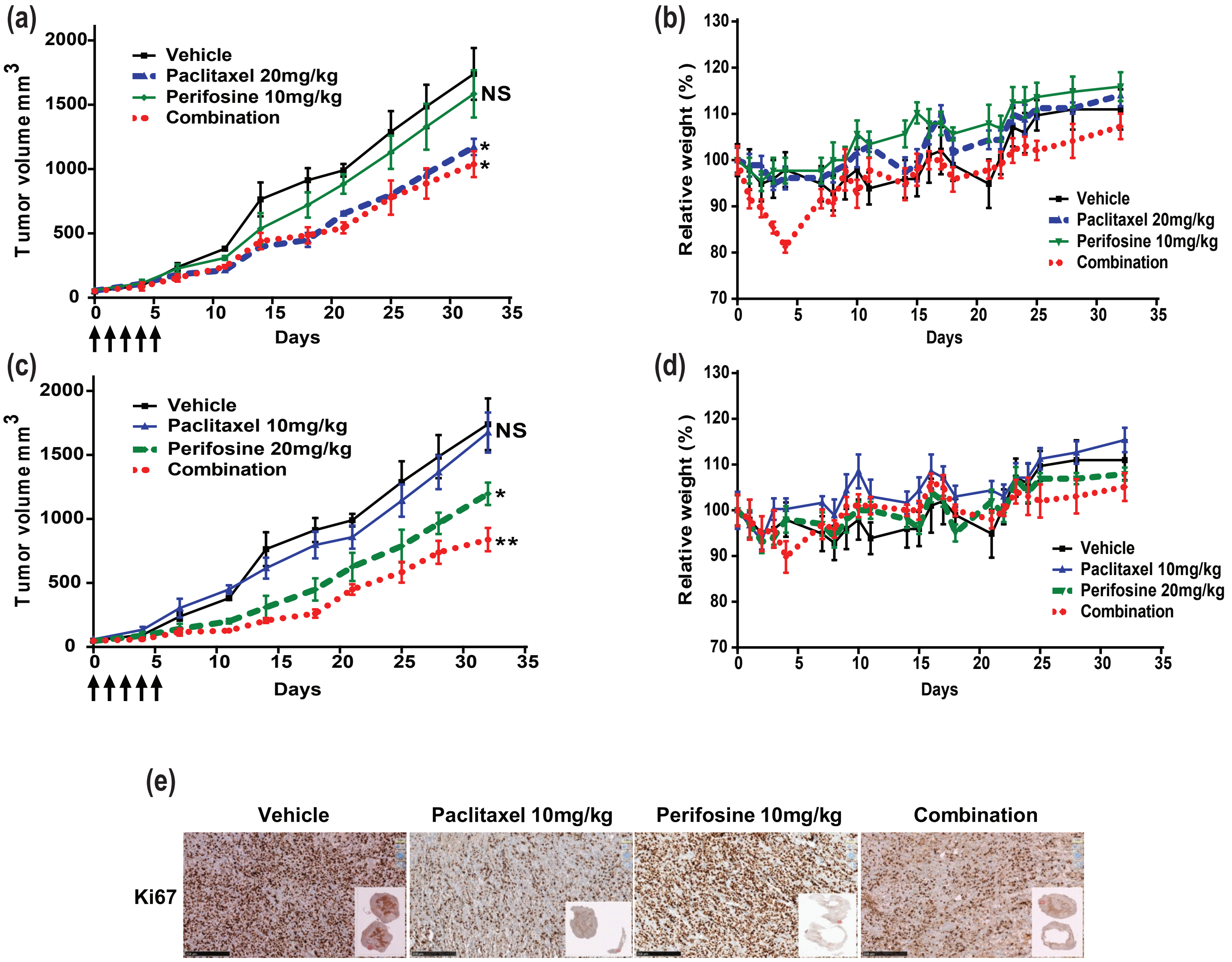

(a)
Tumor volume mm3
Vehicle
Paclitaxel 20mg/kg
Perifosine 10mg/kg
Combination
NS
*
*
2000
1500
1000
500
0
0 5 10 15 20 25 30 35
Days
(b)
Relative weight (%)
130
120
110
100
90
80
70
0 5 10 15 20 25 30 35
Days
Vehicle
Paclitaxel 20mg/kg
Perifosine 10mg/kg
Combination
(c)
Tumor volume mm3
Vehicle
Paclitaxel 10mg/kg
Perifosine 20mg/kg
Combination
NS
*
**
2000
1500
1000
500
0
0 5 10 15 20 25 30 35
Days
(d)
Relative weight (%)
130
120
110
100
90
80
70
0 5 10 15 20 25 30 35
Days
Vehicle
Paclitaxel 10mg/kg
Perifosine 20mg/kg
Combination
(e)
Vehicle
Paclitaxel 10mg/kg
Perifosine 10mg/kg
Combination
Ki67

Supplementary Figure 3: Perifosine was administered by oral gavage 5 days/week throughout the study and paclitaxel was administered either once daily the first five days (n=5) (a-c). Significant differences compared to vehicle. (b-d) Weight of mice was determined. (e) Representative pictures of nuclear proliferation marker Ki67 antibody staining were shown (scale bars 250 µm).

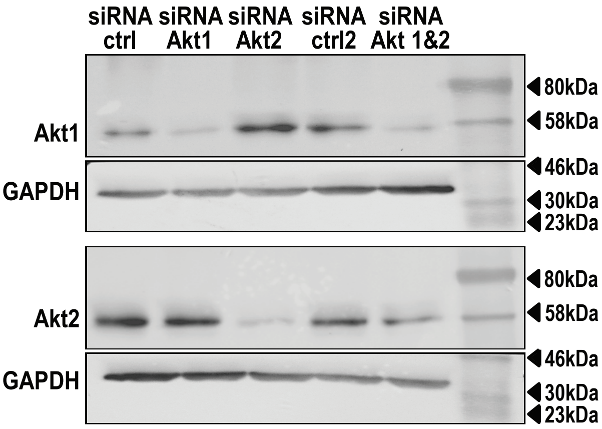

siRNA ctrl
siRNA Akt1
siRNA Akt2
siRNA ctrl2
siRNA Akt 1&2
Akt1
GAPDH
Akt2
GAPDH
80kDa
58kDa
46kDa
30kDa
23kDa
80kDa
58kDa
46kDa
30kDa
23kDa

Supplementary Figure 4: Uncropped blots are presented with levels of Akt1, Akt2 and GAPDH in A549 cells transfected with a control siRNA, Akt1 or Akt2 siRNAs. Samples derive from the same experiment and blots are processed in parallel. Molecular size markers are represented by using ColorPlus Prestained Protein Marker broad range (7-175kDa; New England; BioLabs). Non-overexposure of luminescent signals was guaranteed by using the SynGene Software (saturation tools).

Cell lines	Culture method	Specifications
A549<br>Non-small cell lung cancer	RPMI-1640 medium<br>10 % fetal bovine serum<br>2 mM L-glutamine<br>1 % penicillin/streptomycin	$EGFR^{wt}$
A549/EpoB40<br>Non-small cell lung cancer	RPMI-1640 medium<br>10 % fetal bovine serum<br>2 mM L-glutamine<br>1 % penicillin/streptomycin<br>40 nM patupilone	$EGFR^{wt}$;<br>resistant cell line to paclitaxel derived from A549
H1650<br>Non-small cell lung cancer	RPMI-1640 medium<br>10 % fetal bovine serum<br>2 mM L-glutamine<br>1 % penicillin/streptomycin	EGFR DE746-A750 in exon 19
H1975<br>Non-small cell lung cancer		EGFR L858R in exon 21 and T790M in exon 20 ;<br>resistant cell line to anti-EGFR
HCC827<br>Non-small cell lung cancer		EGFR DE746-A750 in exon 19;<br>resistant cell line to paclitaxel
MDA-MB231<br>Breast cancer		
SK-N-SH<br>Neuroblastoma		
BE(2)C<br>Neuroblastoma	DMEM medium<br>10 % fetal bovine serum<br>2 mM L-glutamine	
BE(2)C/VCR10<br>Neuroblastoma		Resistant cell line to vincristine derived from BE(2)C)
BE(2)C/ADR20<br>Neuroblastoma		Resistant cell line to adriamycin derived from BE(2)C)
U87-MG<br>Glioblastoma	MEM medium<br>10 % fetal bovine serum<br>2 mM L-glutamine	
HEK293T<br>Epithelial embryonic kidney	DMEM medium<br>10 % fetal bovine serum<br>2 mM L-glutamine<br>1 % non-essential amino acids	Non-cancer cell lines
NHDF<br>Normal dermal fibroblast		
HaCat<br>Human keratinocytes	FBM medium	

Supplementary table 1: Cell line characteristics. All cell lines were routinely maintained in culture at 37°C and 5 % CO_2 and were regularly screened and are free from mycoplasma contamination.

Suppliers:

- Invitrogen, France: RPMI-1640, DMEM and MEM media
- Lonza, Levallois-Perret, France: L-glutamine FBM medium and FBS
- Sigma-Aldrich, Saint-Louis, MO, USA: penicillin/streptomycin and patupilone.

	Paclitaxel 50nM	Paclitaxel 100nM	Perifosine
A549/EpoB40	0.333	0.313	1µM
	1.002	0.723	5µM
	0.738	0.297	10µM

Supplementary Table 2: Combination indexes of perifosine and paclitaxel in A549/EpoB40 NSCLC cells. A color code is used to illustrate the different drug interactions: synergism (green) and additivity (grey).
